# Three-dimensional reconstruction of Y-IrNi rhombic dodecahedron nanoframe by STEM/EDS tomography

**DOI:** 10.1186/s42649-023-00092-7

**Published:** 2023-09-21

**Authors:** Taekyung Kim, Yongsang Lee, Yongju Hong, Kwangyeol Lee, Hionsuck Baik

**Affiliations:** 1 Korea Basic Science Institute, Seoul, 02841 Republic of Korea; 2 Department of Chemistry and Research Institute for Natural Science, Korea University, Seoul, 02841 Republic of Korea

**Keywords:** Transmission electron microscopy, Energy dispersive X-ray spectroscopy, Tomography, Three-dimensional reconstruction

## Abstract

The structural analysis of nanocrystals via transmission electron microscopy (TEM) is a valuable technique for the material science field. Recently, two-dimensional images by scanning TEM (STEM) and energy-dispersive X-ray spectroscopy (EDS) have successfully extended to three-dimensional (3D) imaging by tomography. However, despite improving TEM instruments and measurement techniques, detector shadowing, the missing-wedge problem, X-ray absorption effects, etc., significant challenges still remain; therefore, the various required corrections should be considered and applied when performing quantitative tomography. Nonetheless, this 3D reconstruction technique can facilitate active site analysis and the development of nanocatalyst systems, such as water electrolysis and fuel cell. Herein, we present a 3D reconstruction technique to obtain tomograms of IrNi rhombic dodecahedral nanoframes (IrNi-RFs) from STEM and EDS images by applying simultaneous iterative reconstruction technique and total variation minimization algorithms. From characterizing the morphology and spatial chemical composition of the Ir and Ni atoms in the nanoframes, we were able to infer the origin of the physical and catalytic durability of IrNi-RFs. Also, by calculating the surface area and volume of the 3D reconstructed model, we were able to quantify the Ir-to-Ni composition ratio and compare it to the EDS measurement result.

**Supplementary Information:**

The online version contains supplementary material available at 10.1186/s42649-023-00092-7.

## Introduction

Most structural analysis techniques for nanomaterials, such as catalysts, batteries, and semiconductor devices, have been performed by using two-dimensional (2D)-based imaging techniques; in most cases, the information gathered via the 2D methods has been sufficient in deciphering the observed material properties (Park et al. 2018; Kim et al. 2019; Steimle et al. 2020). However, recent advances in nanomaterials for fuel cell, water splitting, etc., have demanded the determination of accurate three-dimensional (3D) structures to reveal the origins of the properties (Wang et al. 2021; Lee et al. 2022a, b). For this purpose, transmission electron microscope (TEM) has become an inevitable tool for the study of nanocrystal and microcrystal structures. In particular, the application of high-angle annular dark-field (HAADF) imaging in scanning TEM (STEM) mode in Cs-corrected TEM has proven to be an invaluable tool due to its high resolution based on the atomic number (Midgley and Weyland 2003; Van Aert et al. 2011; Scott et al. 2012). Furthermore, the recently developed technique of atomic electron tomography has been a powerful method for atomic-level 3D imaging, which can determine the defects, chemical disorder, strains, etc., in nanoparticles (Miao et al. 2016; Lee et al. 2021). However, STEM-based tomography with low-contrast or multiple-component materials with similar atomic numbers often encounters difficulties in the acquisition of clearly differentiated tomograms (Genc et al. 2013).

Tomography using spectral imaging by energy dispersive X-ray spectroscopy (EDS) or electron energy-loss spectroscopy (EELS) has also been investigated over the past few decades; (Yaguchi et al. 2008; Jarausch et al. 2009; Zhong et al. 2018) it has been demonstrated to provide spatially distributed chemical information for nanomaterials, compensating for the aforementioned limitation of STEM tomography. Furthermore, the advances in EDS detector configuration and holder design have allowed for the simple and efficient operation of EDS mapping, while EELS suffered from the inelastic scattering artifacts by sample thickness variation (Yedra et al. 2012). However, the EDS- and EELS-based imaging techniques typically require a longer acquisition time than the STEM technique to obtain the tilt series, which tends to result in unnecessary contamination or damage to the target materials. In addition, tomography results tend to suffer from the so-called “missing wedge” problem in tomography results in more complicated artifacts or elongation along the direction of missing angles in EDS tomography, which would lead to the difficult interpretation of the reconstruction result (Midgley and Dunin-Borkowski 2009).

In this work, we present a 3D reconstruction technique with STEM and EDS tilt series of IrNi rhombic dodecahedral nanoframes (IrNi-RFs) that was used as an electrocatalyst for oxygen evolution reaction (Jin et al. 2017) to investigate the structural characteristics and spatial distribution of the elements in a nanoframe. We first compared the representative algorithms, such as direct Fourier method, simple back projection, the simultaneous iterative reconstruction technique (SIRT), and total variation minimization (TV-M), and optimized the reconstruction condition with SIRT and TV-M by changing the iteration numbers. From the STEM tomography by SIRT and TV-M algorithm, we could clearly observe the rhombic dodecahedral nanoframe morphology of IrNi-RFs, which revealed the characteristic rotational symmetries of the rhombic dodecahedron. EDS tomography based on the use of 2D elemental mapping images of Ir and Ni atoms provided the spatial distribution information in the nanoframe; Ir was distributed over the nanoframe, and Ni was mainly located at the vertices of the nanoframe. Additionally, we quantified the ratio of Ir to Ni atoms by calculating the reconstructed volume of each element. This work clearly demonstrates the great potential of three-dimensional analysis of nanocatalysts in understanding the structural features most pertinent to catalytic performance and in further applying this knowledge in designing more advanced nanocatalysts.

## Materials and methods

### Materials

Ir(acac)_3_ (97%), Pt(acac)_2_ (97%), YCl_3_ (99.99%), hexadecyltrimethylammonium chloride (> 98%), trioctylphosphine oxide (90%), and oleylamine (98%) were purchased from Sigma-Aldrich. NiCl_2_ (98%) was purchased from Alfa-Aesar. All reagents were used as received without further purification.

### Preparation of rhombic dodecahedral Y-IrNi nanoparticles

The rhombic dodecahedral IrNi nanoparticles (IrNi-RPs) were synthesized as described in the literature (Jin et al. 2017). In brief, a slurry of YCl_3_ (0.05 mmol), Ir(acac)_3_ (0.006 mmol), and NiCl_2_ (0.05 mmol) in oleylamine (12 mmol) were evacuated at 100 °C for 10 min and charged with 1 atm of Ar gas. Then, the slurry, equipped with a bubbler, was heated at 280 °C in the oil bath at a rate of 10 °C per min. After it reached 280 °C, the further reaction continued at that temperature for 35 min. After the solution was cooled to room temperature, the reaction mixture was washed several times with toluene and ethanol to remove remained substances and collected by centrifugation.

### Preparation of rhombic dodecahedral Y-IrNi nanoframes

The IrNi-RFs were synthesized from the IrNi-RPs by applying a nital solution, which is an acidic solution of ethanol and nitric acid, to etch out the core Ni component (Jin et al. 2017). Generally, to obtain an IrNi-RF, 10 to 20 mg of IrNi-RPs was dispersed in 15 mL of nital solution by applying a magnetic stirrer for 3 h at 40 °C. Afterward, the product was collected by applying centrifugation after washing it several times with ethanol.

### TEM experiments and tomography

**Scheme 1:**
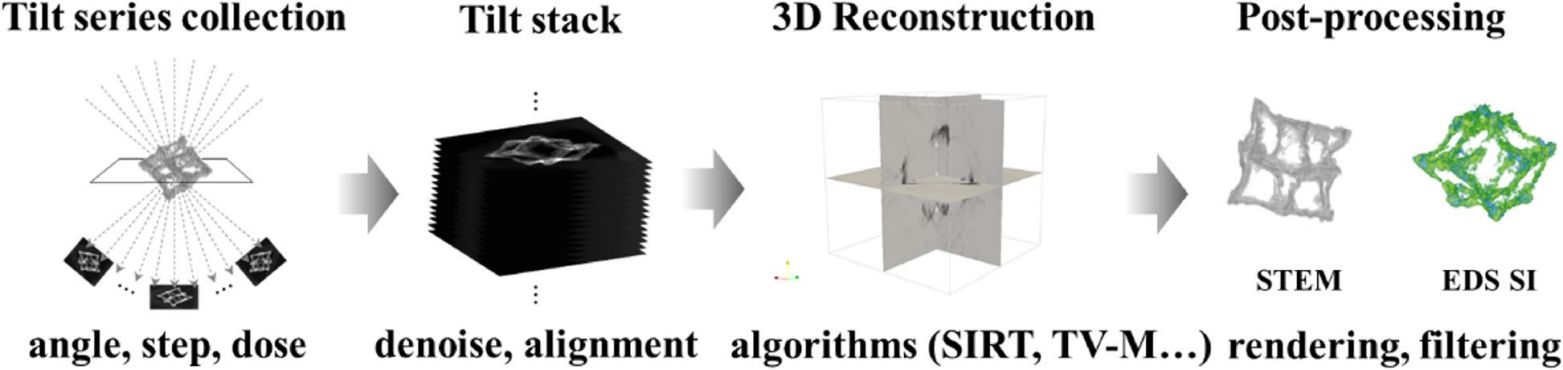
Schematic illustration of STEM and EDS tomography process

To obtain the STEM and EDS spectrum images, FEI Double Cs-corrected Titan Themis transmission electron microscope instrument equipped with Super-X EDX detector with an X-FEG module was operated at 300 kV. Samples were loaded in a Fischione Instrument Model 2050 tomography holder. To acquire the tilt series of the STEM and EDS spectrum images, Velox software (Thermo Fisher Scientific) was used, and each tilt series was collected from − 50° to + 70° in 5° steps. The HAADF-STEM and EDS images were simultaneously acquired under the conditions of a magnification of 543 kX, with 1024 × 1024 pixels (pixel size = 180 pm) and a beam current of 342 pA; for each tilt angle, 30 frames of images were collected at a dwell time of 5 μs. Tilt series alignment was carried out by first applying cross-correlation and then tilt-axis rotation/shift alignment; the 3D reconstruction via the SIRT and TV-M algorithm was performed by using tomviz software (Levin et al. 2018) (the resolution of input images was adjusted to 512 × 512 pixels). The regularization parameter for TV-M was used as default value in the tomviz software. Avizo software (Thermo Fisher Scientific) was used for post-treatment, visualization, and quantification. The summarized tomographic process is illustrated in Scheme 1.

## Results and discussion

### Characterization of IrNi-RPs and IrNi-RFs

**Fig. 1:**
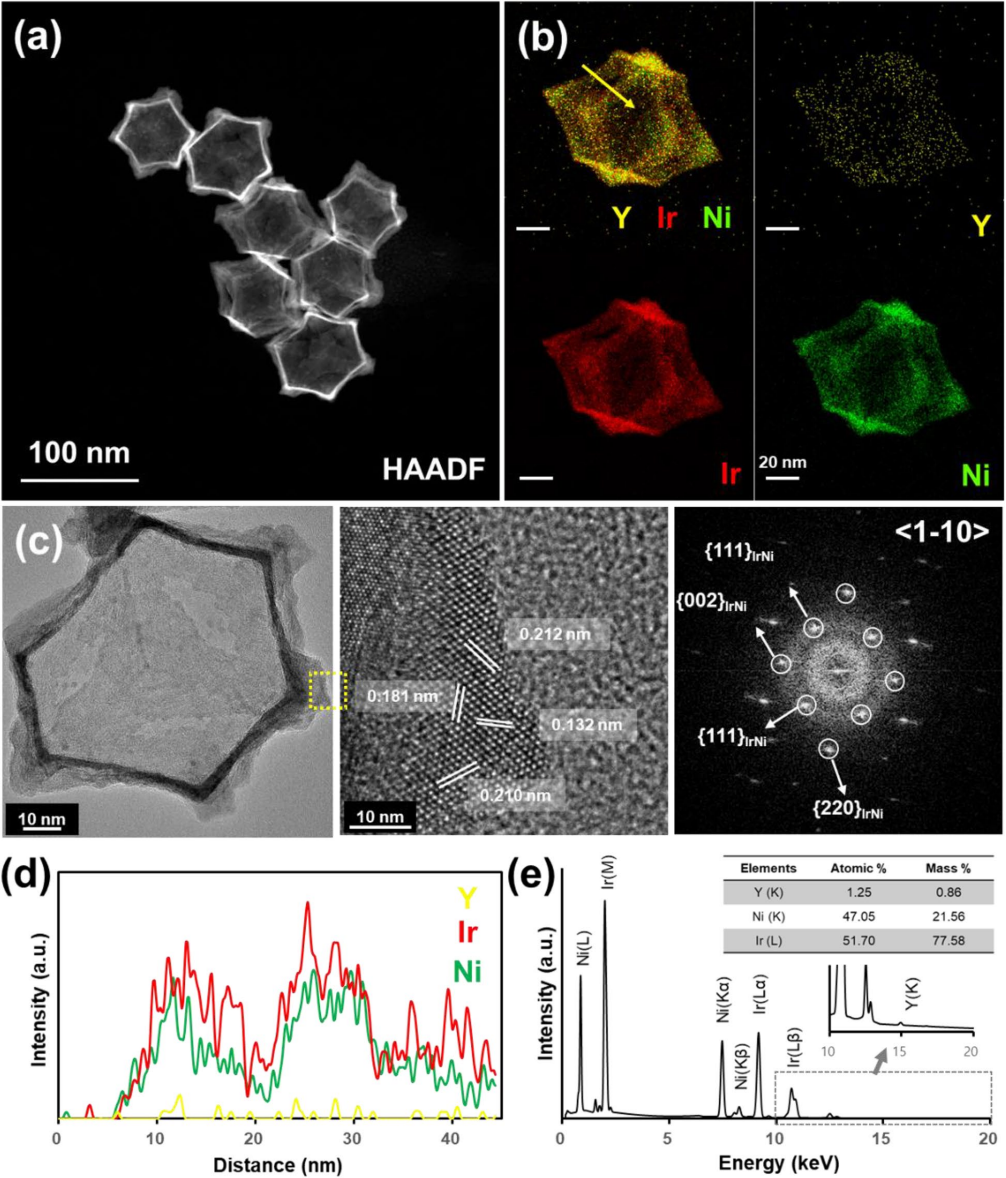
**a** HAADF-STEM, **b** EDS elemental mapping images of IrNi-RF with each element and merged image (Y: yellow, Ir: red, Ni: green), **c** high resolution TEM images of IrNi-RF with enlarged image and corresponding FFT pattern along the $<1\overline{1 }0>$ zone axis. **d** Line profile of Y, Ir, and Ni atoms by EDS (yellow arrow in (**b**)) and **e** EDS spectrum with quantification table by element

To confirm whether the structure and composition of the target system, IrNi-RFs, were appropriately prepared, the synthesized nanoparticles were investigated by using TEM analysis. A representative STEM image of the IrNi-RPs is shown in Fig. S1a. The characteristic structural shape of rhombic dodecahedrons could be clearly observed. The EDS elemental mapping results (Fig. S1b) and line profile (inset in Fig. S1a) indicate that the Ir atoms located on the surface of the dodecahedral Ni core nanoparticles as a core–shell structure, as well as a very small amount of yttrium (Y) atoms, were dispersed throughout the IrNi-RP sample. High-resolution STEM images of IrNi-RP samples are shown in Fig. 1c and d; we observed lattice spacings of 0.215, 0.208, and 0.183 nm on the faces and edges of the IrNi-RP samples at the zone axis of $<1\overline{1}0>$, which corresponded to {111}IrNi and {002}IrNi facets (cif #1,522,777 from crystallography open database), respectively, confirming that face-centered cubic IrNi alloy crystals had successfully formed.

Figure 1a shows the structure of an IrNi-RF after the Ni core was etched out from the IrNi-RP sample by using the nital solution (see the Materials and methods section for the details). The brighter edge contrast (as compared to Fig. S1a) indicates that the core part had been removed and the framework remained intact. Based on the elemental mapping (Fig. 1b) and line profile results (Fig. 1d), the Ir and Ni atoms seem to have been uniformly distributed over the nanoscale framework with an Ir to Ni ratio of 51.7:47.05. The high-resolution TEM images and corresponding fast Fourier transform pattern show that the lattice spacings of 0.212, 0.181, and 0.132 nm correspond to {111}IrNi, {002}IrNi, and {220}IrNi, respectively (Fig. 1c). This confirmed that IrNi-RFs had properly formed, that their crystal structure basically was consistent with that of IrNi-RPs, and that etching can be effectively applied to obtain the frame structure without compromising the nature of the original particles.

### STEM and EDS tomography

As seen in Scheme 1, the process of tomography primarily consists of four steps. First, the tilt series at each tilted angle must be collected. The tilt angle range, angle step, and electron beam dose should be considered to acquire the best raw data. In general, wider tilt angle ranges, smaller angle steps, and higher electron beam doses resulted in better outcomes. However, generally, the available maximum tilt angle range is − 70° to + 70° and the angle step range was 2–5°, as limited by the TEM equipment and time efficiency. This tends to create the missing-wedge problem when reconstructing 3D structures of a target system (Midgley and Weyland 2003). Unlike STEM images, when acquiring EDS spectral images (SIs), a longer duration or higher electron beam current could result in higher intensity counts, which would result in more precisely captured features of the target particles. However, this tends to damage the target nanoparticles; thus, the electron beam dose should be optimized.

As shown in Figs. S2–S4, the tilt series of 25 projections of HAADF-STEM images and EDS SIs of the Ir and Ni atoms of IrNi-RF samples were taken in the range of − 50° to + 70°, with an angle step of 5°, using the aberration-corrected TEM. A beam current of 342 pA was optimized to minimize damage to the IrNi-RFs due to electron beam exposure when capturing the EDS image and Fig. S5 exhibits HAADF-STEM images of IrNi-RF from all projections obtained before and after experiment to confirm the beam damage. Figure S6 shows the EDS results from 15 × 15 pixels with the projection at + 50°. When exporting the EDS SIs of the Ir and Ni elements at each tilt angle, an average fitting area of 15 × 15 pixels (= 2.7 × 2.7 nm^2^) was applied for each element because a 1 × 1 pixel size could not provide enough counts to obtain reliable EDS SIs. For the EDS maps, Ir-M and Ni-L lines and Ir-L and Ni-K lines were used for EDS SIs, which are not very close to each other. As usual, EDS setup shows the angular dependency among the electron counts in the EDS images; thus, the intensity normalization process was carried out before the 3D reconstruction.

**Fig. 2:**
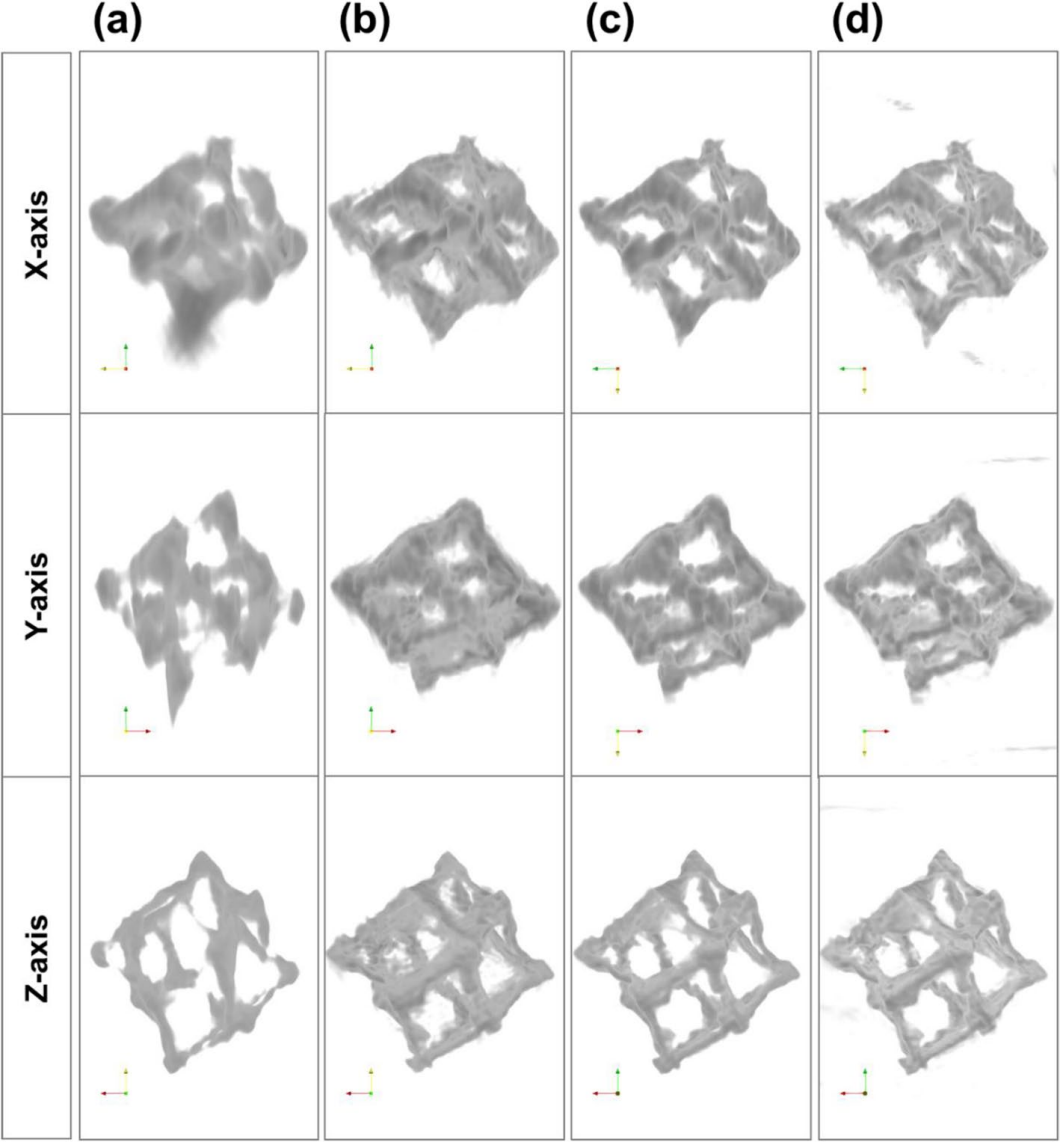
3D reconstruction result by (**a**) simple back projection, (**b**) direct Fourier, (**c**) SIRT, and (**d**) TV-M algorithm via STEM tomography

As the first step of 3D reconstruction, the obtained tilt series were aligned by performing the cross-correlations between neighboring images with the degree of shifts and tilt axis rotation; shift alignment was also applied. These alignment methods have been described in the previous reports (Bosman et al. 2006; Uusimäki et al. 2013; Schwartz et al. 2022). The aforementioned alignment process was performed by using the HAADF-STEM tilt series first; then, the alignment parameters were exported and applied to the EDS SI because the sparse intensity of EDS SIs tends to lead to undesirable alignment results. In this study, the optimized 3D reconstruction process was implemented by applying the aligned HAADF-STEM and EDS SIs obtained via the SIRT, widely known efficient iterative method, and TV-M algorithm, which served to alleviate the missing-wedge problem (Goris et al. 2012). The aim of both algorithms is to find a solution to satisfy the equation of Ax = b, where A, b, and x represent the scanning process, projections, and 3D images, respectively. This can be accomplished by minimizing the projection distance (b − Ax) and total variation. In the case of TV-M, the regularization parameter is also crucial in determining the quality of 3D reconstruction. In this work, we utilized the default value provided by the software (Goris et al. 2012). Additionally, before the selection of SIRT and TV-M, a few other algorithms, i.e., simple back projection, direct Fourier method, the SIRT, and TV-M, were also evaluated to find the suitable representatives for 3D reconstruction with the aligned HAADF-STEM tilt stack. As can be seen in Fig. 2, the reconstruction via the SIRT and TV-M resulted in higher-quality images of the structures as compared to simple back projection and direct Fourier reconstruction, despite the the latter group’s efficiency of calculation time.

**Fig. 3:**
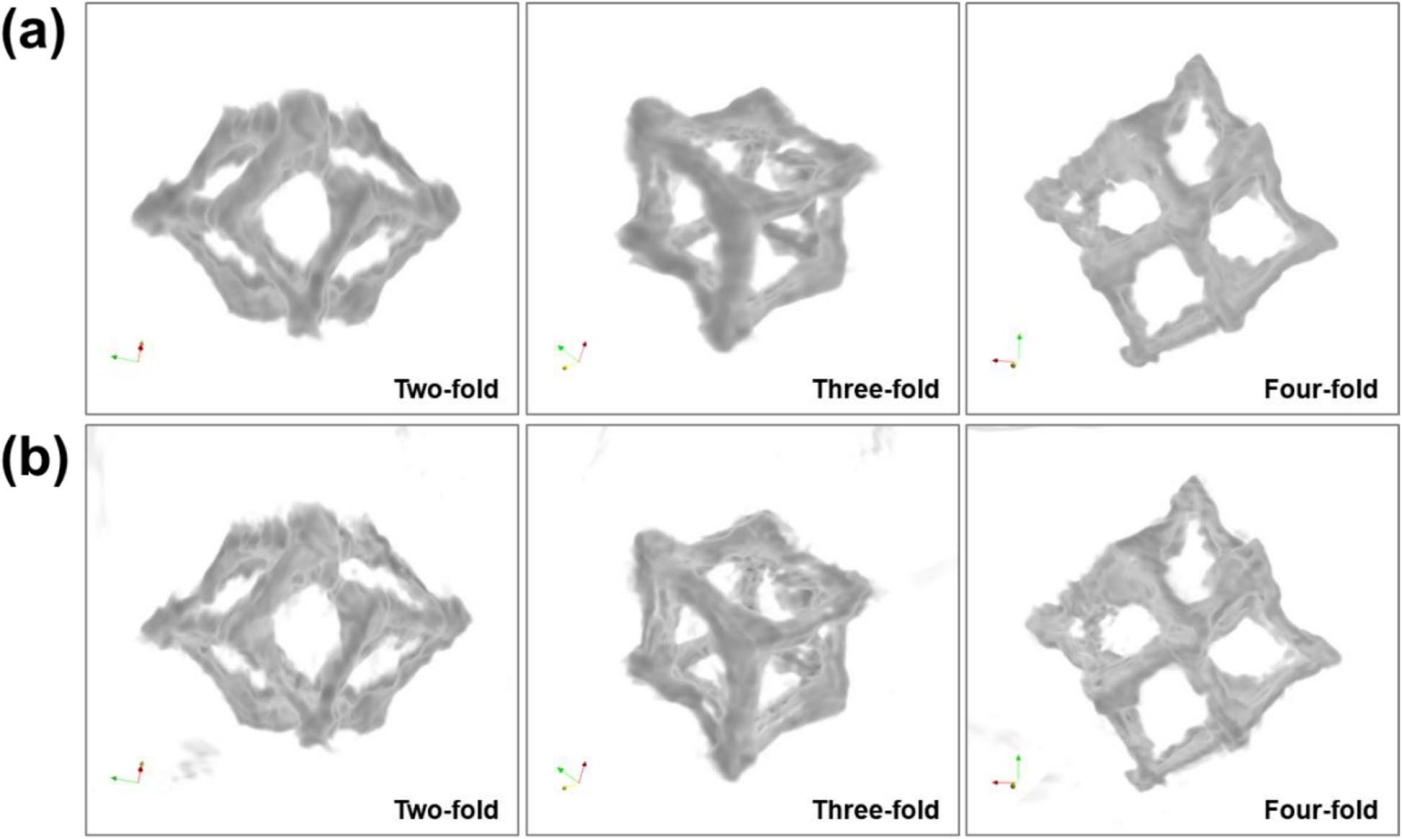
Comparison of the optimized 3D reconstruction result along the symmetry axes by (**a**) SIRT and (**b**) TV-M via STEM tomography at the 50 times of iteration

Figure 3 exhibits the 3D reconstruction of an IrNi-RF sample that has been optimized by applying the SIRT and TV-M (iteration number = 50) after volume rendering with the same threshold values. For better imaging and comparison, Gaussian blurring with a sigma of 2.0 was applied to each sinogram before volume rendering. Initially, the IrNi-RF sample resulting from the SIRT was found to have a smoother surface than that obtained via TV-M. When considering 25 projections of HAADF-STEM images with the aforementioned results, this difference can be explained by the fact that TV-M was able to describe the details of a nanoparticle’s characteristic features, whereas the SIRT simplified the structure of the nanoparticle, even though Gaussian filtering was applied. Although the missing wedge may have had considerable influence (the tilt series was obtained from − 50° to + 70°), both the SIRT and TV-M yielded well-constructed framework features. In each tomogram, the characteristic two-, three-, and four-fold symmetries of the rhombic dodecahedron (Kim et al. 2020) were found within the skeletal framework, indicating that the 3D reconstruction for the IrNi-RF was performed appropriately by both algorithms. The nanoparticles with the skeletal frameworks were observed to have curved and bent edges due to their intrinsic weakness, deformation that occurred during the preparation process, or accumulated electron beam damage during observation. As can be seen in Fig. 3, the STEM-based reconstruction reflected the aforementioned phenomena on the structure of the IrNi-RF.

Typically, the quality of reconstructed images improves as the number of iterations increases. Additionally, the noise in images may increase (Heidari Mezerji et al. 2011). To investigate and compare the influence of the number of iterations in the SIRT and TV-M systems, the reconstruction was performed with 10, 20, 30, 50, and 100 iterations for each algorithm (Figs. S7 and S8). In the case of the SIRT algorithm, as the number of iterations increased from 10 to 100, an apparent image quality enhancement effect could be confirmed (see the blue, green, and orange dotted boxes in Fig. S6). In detail, regarding the results for 30 iterations, most of the features of the IrNi-RF seem to be similar to those for 50 and 100 iterations; additionally, it was difficult to find any significant improvement after 50 iterations, which indicates that the SIRT-based 3D reconstructed structure of IrNi-RFs converged after 50 of iterations. Additionally, this result is consistent with the previously reported work (Heidari Mezerji et al. 2011). The IrNi-RF reconstruction via the TV-M algorithm was also performed using different numbers of iterations. Intriguingly, TV-M did not result in any iteration-dependent substantial changes in the reconstructed IrNi-RF structure, as shown in Fig. S8. Rather, because an increase in noise or the number of artifacts degraded the quality of the reconstructed image, it seems that the number of iterations in the calculation via the TV-M algorithm should be determined by trial and error. In this study, the number of iterations for the TV-M algorithm was determined to be 50, because this was the point at which the artifacts began distorting the reconstructed images.

**Fig. 4:**
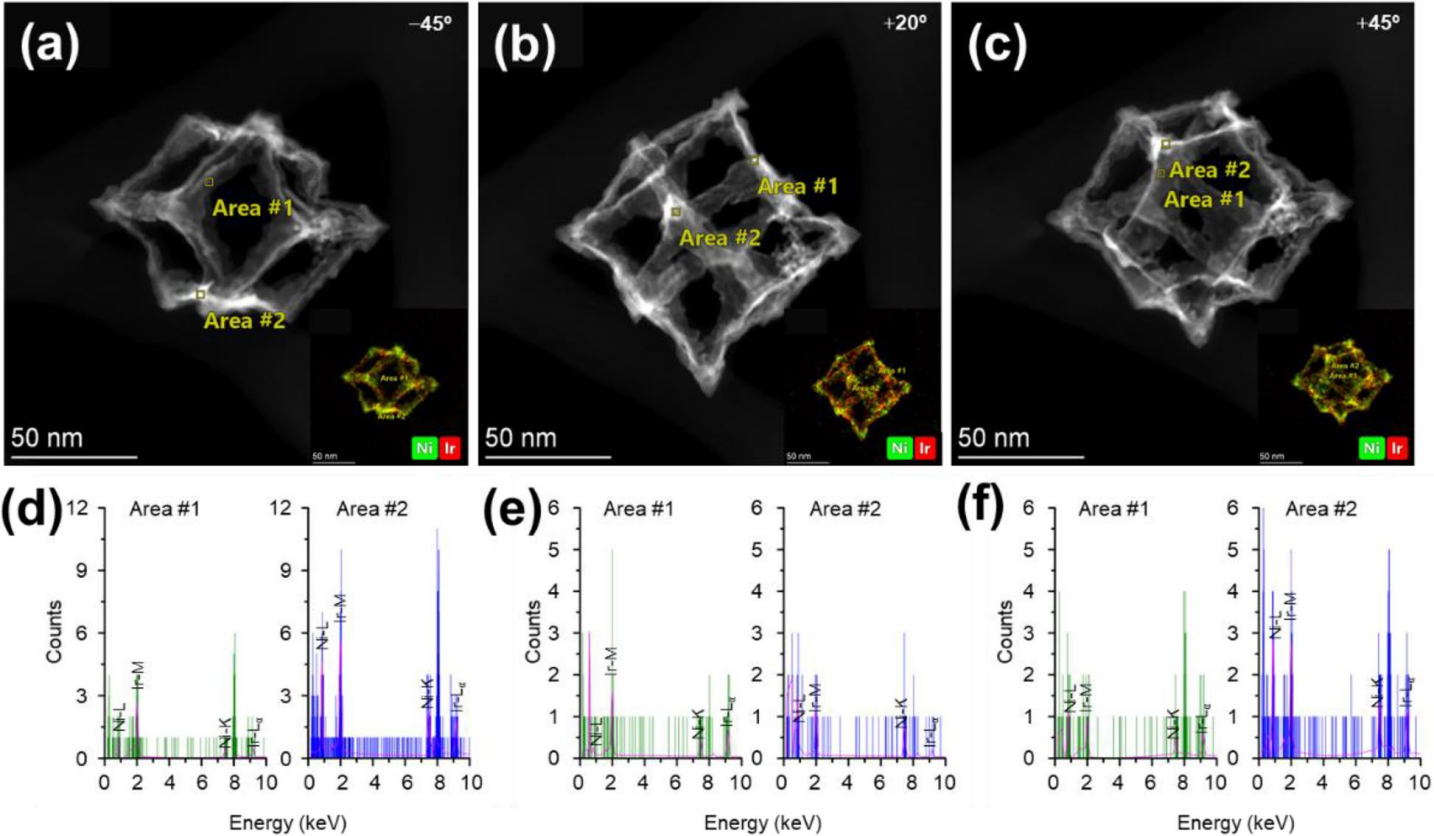
HAADF-STTEM images at tile angle of (**a**) − 45°, **b** + 20°, and (**c**) + 45° with merged EDS mapping of Ir (red) and Ni (green) atoms in inset. **d**, **e**, and **f** are the corresponding EDS spectra of Ir and Ni elements at the location of Area #1 (green) and Area #2 (blue) in each HAADF-STEM image

**Fig. 5:**
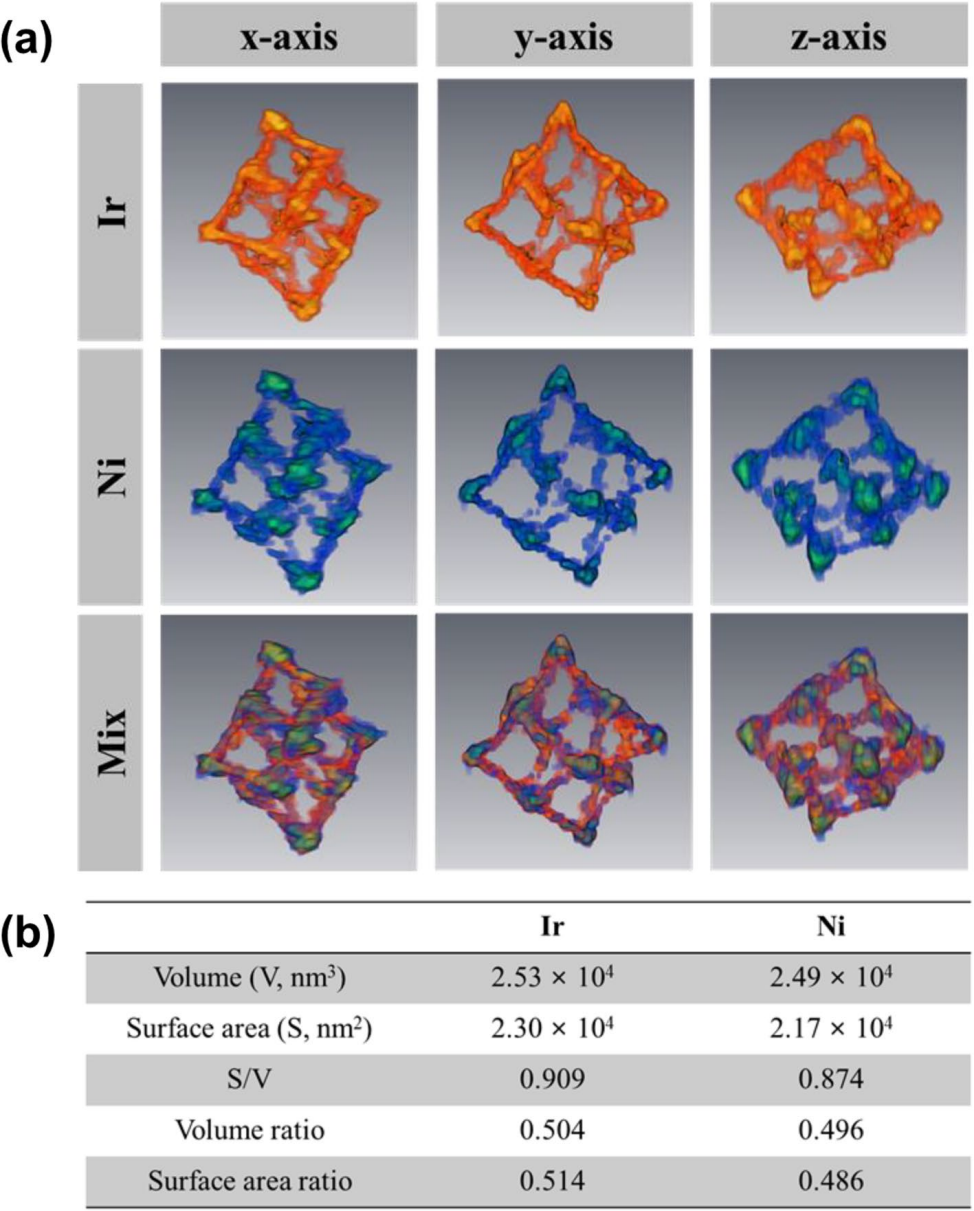
**a** 3D reconstruction result by Ir and Ni EDS spectral images via TV-M algorithm and (**b**) calculation result of volume and surface area for each element with reconstruction result (**a**)

According to the previously reported works, 2D elemental EDS mapping following IrNi-RP etching results in Ir and Ni atoms that are uniformly distributed throughout the skeletal frameworks (Jin et al. 2017). In addition, our 2D EDS elemental mapping analysis initially seemed to yield the same result as that shown in Fig. 1b. Figure 4 shows the distribution of Ir and Ni atoms at the edges (Area #1) and vertices (Area #2) based on the EDS images at three tilt angles, i.e., − 45°, + 20°, and + 45°. The intensities of the Ir and Ni at the edges and vertices tended to increase in the direction of the edges to the vertices; this was observed for all tilt angles, indicating that the Ir and Ni atoms were uniformly distributed across the nanoscale framework. Therefore, we expected the results of 3D reconstruction via EDS tomography to reveal a similar result. Based on the aforementioned STEM tomography results, the 3D reconstruction of EDS SIs of Ir and Ni atoms was attempted via SIRT and TV-M algorithms; the numbers of iterations applied for the SIRT and TV-M were 50 and 10, respectively. In detail, as shown in Fig. S9, both algorithms yielded good 3D reconstructions from EDS SIs; additionally, the entire shapes were similar to those observed as a result of STEM tomography in Fig. 3. The Ir and Ni atoms were observed to exist across the nanoframework, and this observation is consistent with the expectation based on the 2D EDS mapping results. However, it can be seen that the Ni atoms were more located near the vertices of the IrNi-RFs than the Ir atoms. That is, the Ni atoms tended to primarily exist at the vertices after the etching process; this may be supported by the relatively low intensity counts of Ni atoms at the edge positions as compared to Ir atoms (Fig. 4). In a previous report, Ni was shown to contribute to enhancing the stability of the nanostructure owing to its unchanged oxidation state (Jin et al. 2017). Based on the aforementioned results, we expect that the occurrence of Ni distribution at the vertices without a change in oxidation state may have fortified the nanostructure and functioned as one of the sources of the enhancement of catalytic durability for IrNi-RFs in the previous study. In EDS, X-ray intensities are proportional to the probabilities of created X-ray from the samples (Haberfehlner et al. 2014) and a relatively large amount of atoms in a particular space of a sample could result in higher intensities than others; therefore, the reconstructed volume (or voxels) might contain the information for quantitative comparison among the elements of a sample. To quantify the amounts of Ir and Ni atoms, we calculated the volumes of the reconstructed models by using Avizo software. The median filter was applied to reduce the noise, and a dilation and erosion process was applied to remove the minor artifacts from the reconstructed models. Following the treatments, the reconstructed volumes of Ir and Ni were calculated. Figure 5a and b show the optimized Ir and Ni 3D images and volumes, respectively. The total volumes of Ir and Ni in the IrNi-RF were 2.35 × 10^4^ and 2.49 × 10^4^ nm^3^, respectively, and the corresponding volume proportions were 0.504 (Ir) and 0.496 (Ni). Using the EDS-based quantification results as a reference, the aforementioned proportions were similar to the atomic proportions of Ir and Ni, which were 51.70 and 47. 05%, respectively.

## Conclusion

In conclusion, STEM and EDS tomography successfully revealed the characteristic 3D structure and compositional variations of IrNi-RFs; particularly, both the SIRT and TV-M algorithms yielded reasonable reconstruction results when applied for STEM and EDS tomography. In the framework structure, the interpretation of the 2D STEM and EDS mapping images is ambiguous because several skeletal features would be concurrently projected all the time. However, in this study, STEM tomography was demonstrated to clearly provide the morphology of the rhombic dodecahedral framework; furthermore, EDS tomography based on 2D elemental mapping images of Ir and Ni atoms not only provided the structural features, but also the spatial information regarding the composition of the nanoframework. Based on this, we could understand that Ni atoms might serve to form a stable nanostructure and thereby improve catalytic performance. EDS tomography was also found to enable quantification of the elemental composition of IrNi-RFs through the calculation of the reconstructed volume of each element; the results were comparable to the conventional EDS data. These results clearly show that 3D STEM/EDS tomography holds a great promise in material discovery in important application fields such as electrochemical energy conversion and storage.

### Supplementary Information

**Additional file 1: Figures S1-Figure S8.**

## Data Availability

Not applicable.
