# Supplementary material for: Three-dimensional reconstruction of Y-IrNi rhombic dodecahedron nanoframe by STEM/EDS tomography
Source: Appl Microsc. 2023 Sep 21;53:9. doi: 10.1186/s42649-023-00092-7 (PMC10511395; doi:10.1186/s42649-023-00092-7)
Supplement: Supplementary file 1 — Additional file 1: Figures S1-Figure S8. [file 42649_2023_92_MOESM1_ESM.pdf]

Supporting Information

Three-dimensional reconstruction of Y-IrNi rhombic dodecahedron nanoframe by STEM/EDS tomography

Taekyung Kim[1], Yongsang Lee[1], Yongju Hong[2], Kwangyeol Lee[2], and Hionsuck Baik[1,*]

[1]*Korea Basic Science Institute (KBSI), Seoul 02841, Republic of Korea*

[2]*Department of Chemistry and Research Institute for Natural Science, Korea University, Seoul 02841, Republic of Korea*

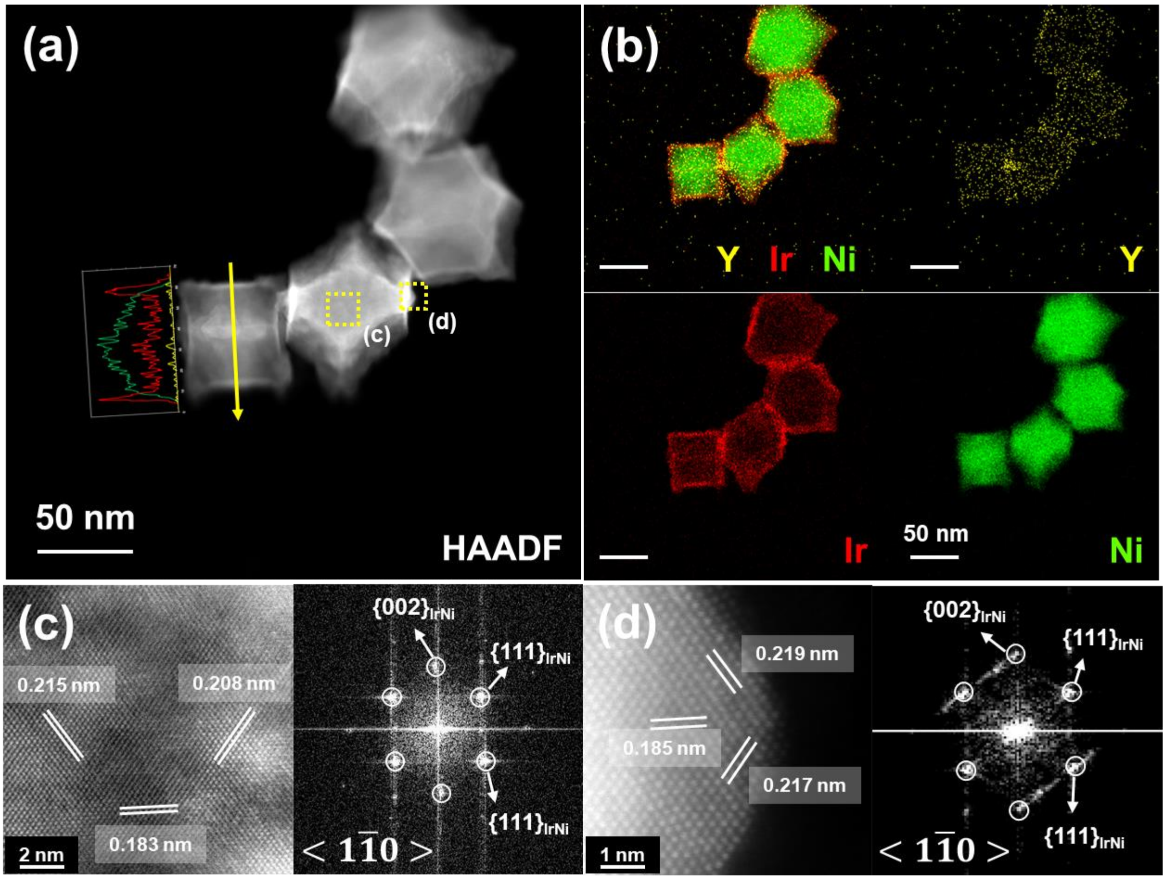

(e)

Elements	Atomic %	Mass %
Y (K)	0.89	1.19
Ni (K)	93.28	82.03
Ir (L)	5.83	0.86

Fig. S1 (a) HAADF-STEM with EDS elemental line profile (Y: yellow, Ir: red, Ni: green), (b) EDS elemental mapping images of IrNi-RP with each element and merged image, (c) and (d) enlarged high resolution STEM images of IrNi-RP with corresponding FFT pattern along the $< 1\bar{1}0 >$ zone axis, and (e) EDS quantification table by composition.

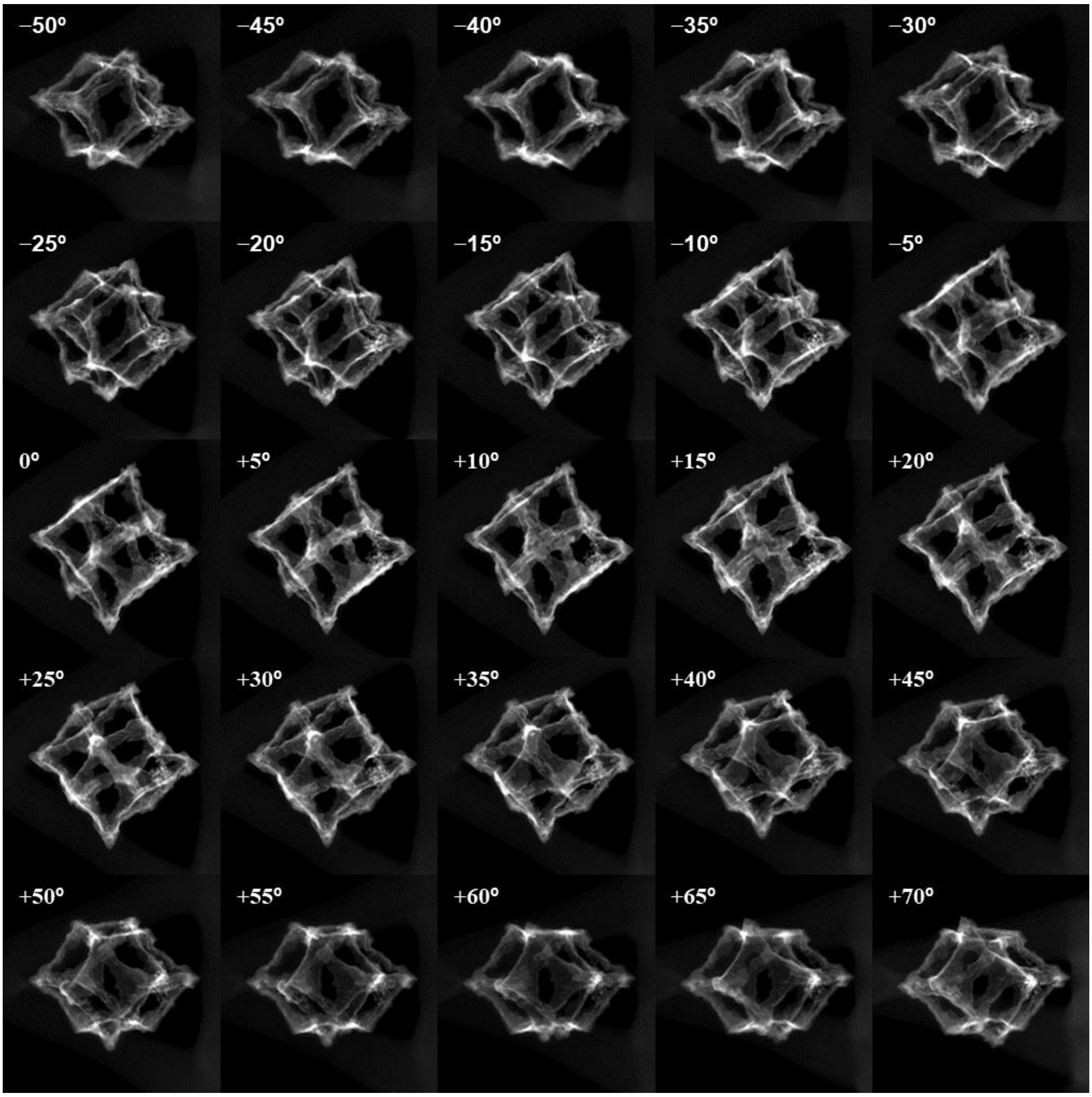

Fig. S2 HAADF-STEM tilt series of IrNi-RF taken from −50° to +70° of rotation angles with 5° of step.

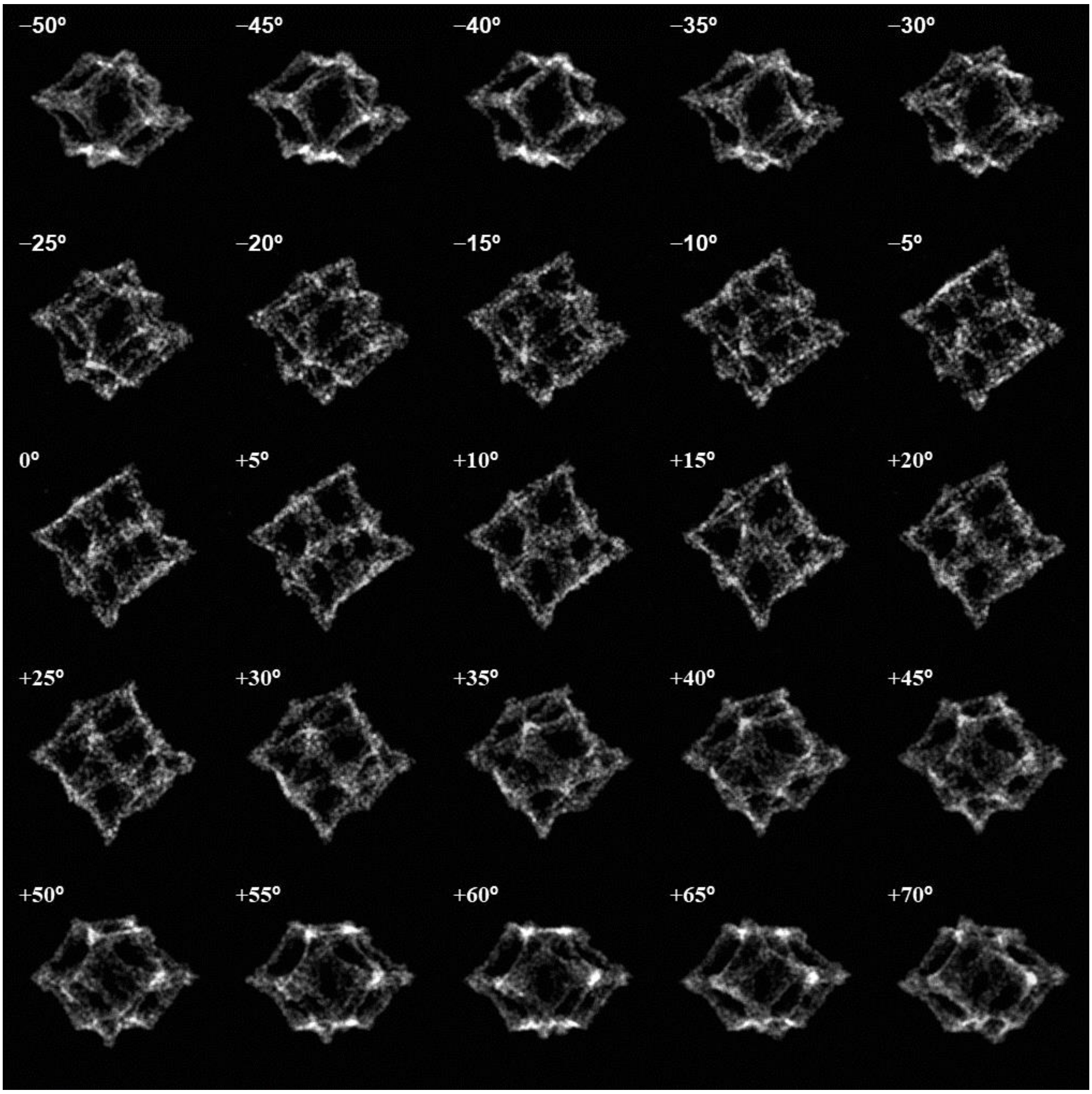

Fig. S3 EDS elemental mapping tilt series of Ir for IrNi-RF taken from −50° to +70° of rotation angles with 5° of step after conversion 8bit scale.

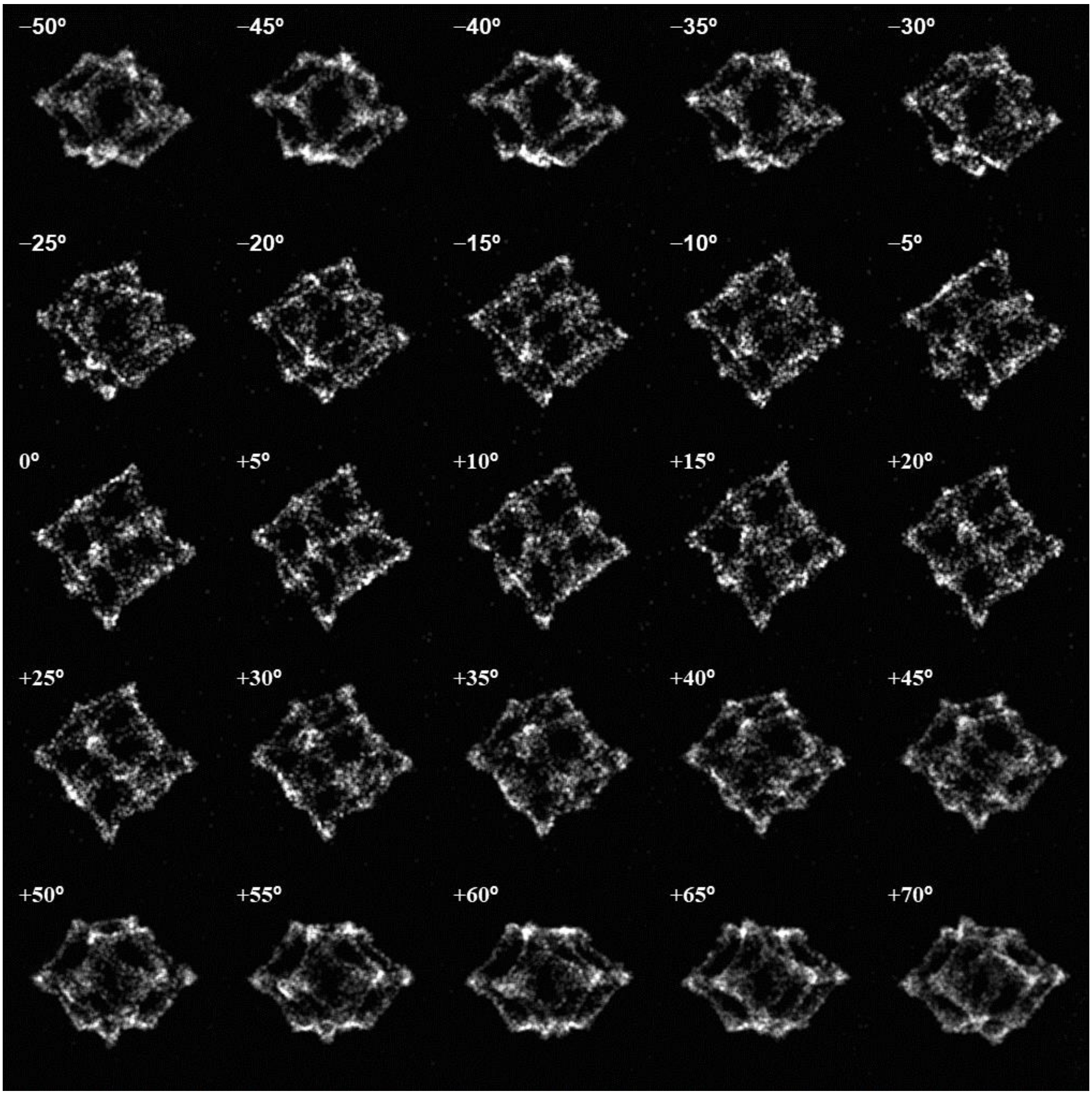

Fig. S4 EDS elemental mapping tilt series of Ni for IrNi-RF taken from −50° to +70° of rotation angles with 5° of step after conversion 8bit scale.

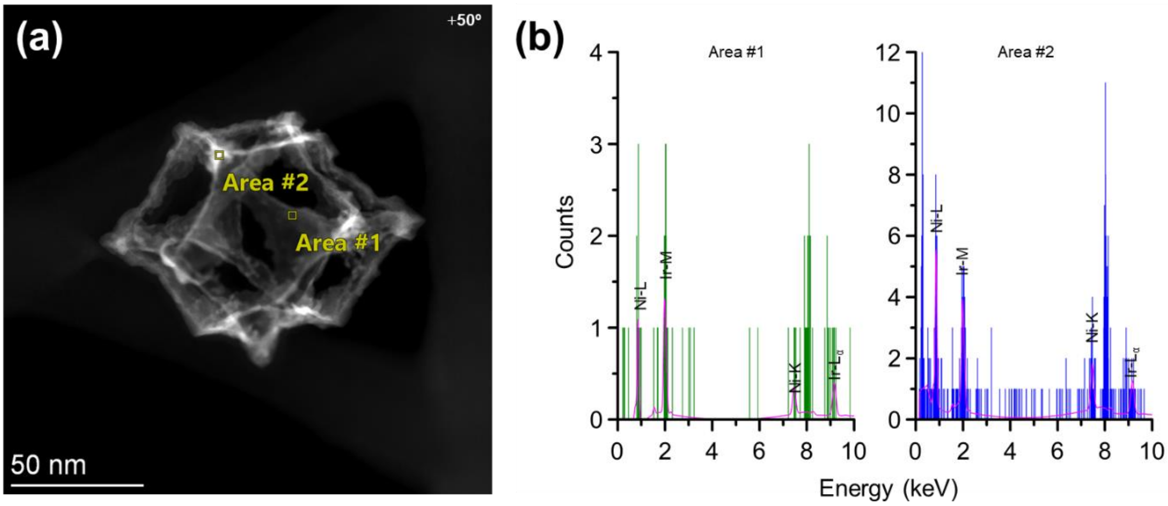

Fig. S5 (a) HAADF-STEM image of IrNi-RF at +50° (b) EDS spectra of Ir and Ni atoms at the location Area #1 and Area #2 in (a). The area of Area #1 and Area #2 is both 15 × 15 pixels.

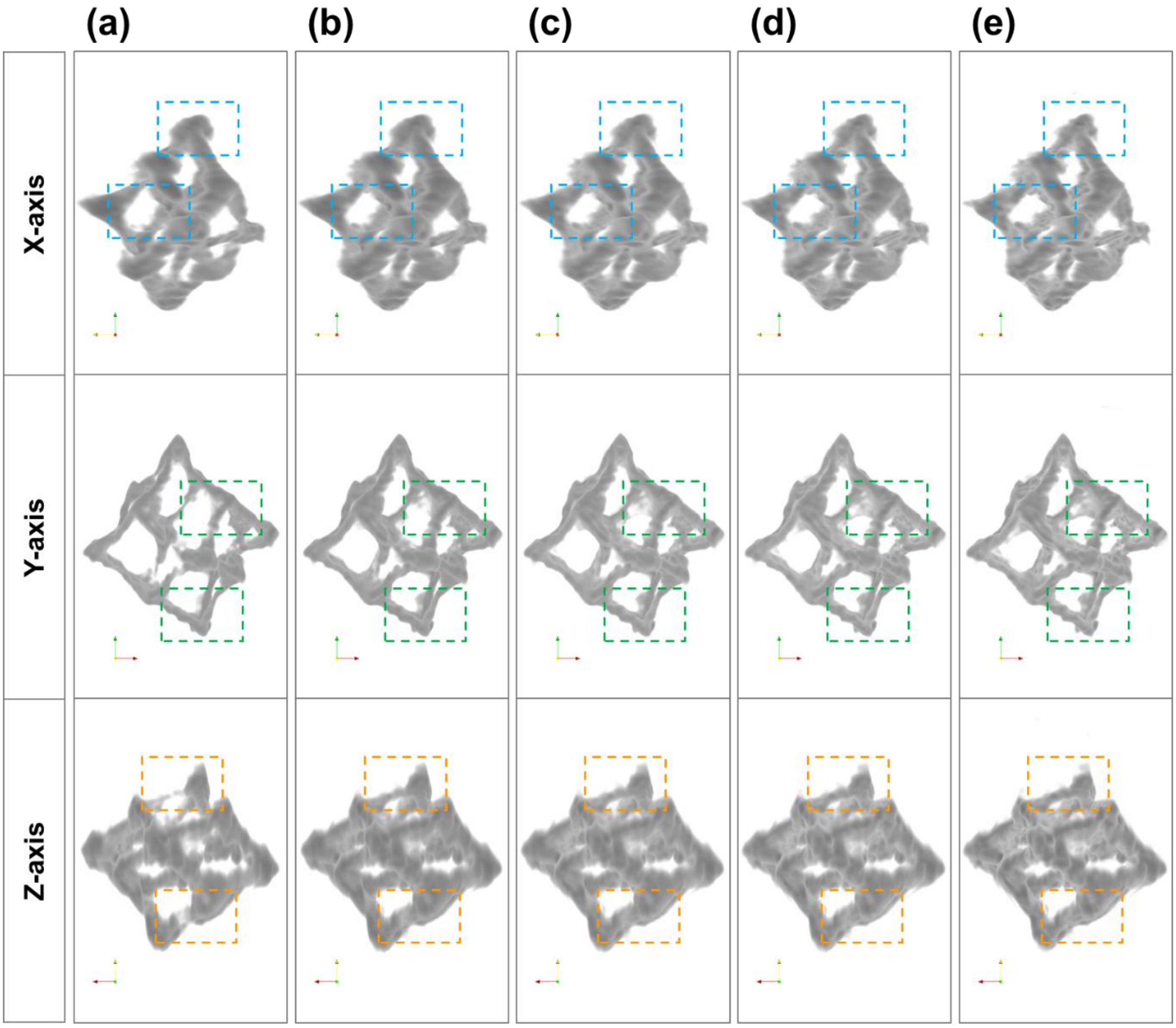

Fig. S6 Comparison of reconstruction result by iteration numbers for SIRT algorithm (a) 10, (b) 20, (c) 30, (d) 50, and (e) 100 times.

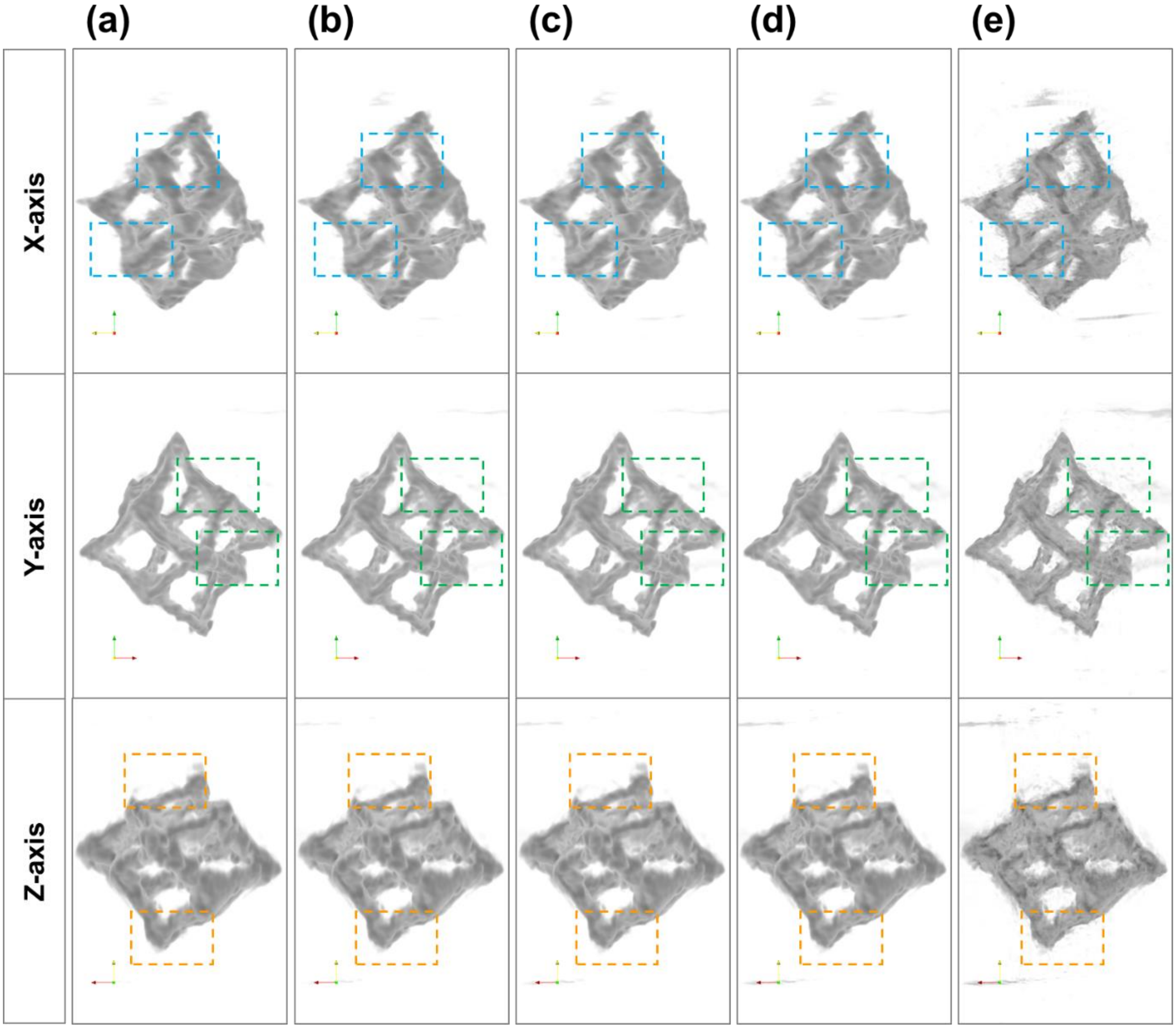

Fig. S7 Comparison of reconstruction result by iteration numbers for TV-M algorithm (a) 10, (b) 20, (c) 30, (d) 50, and (e) 100 times.

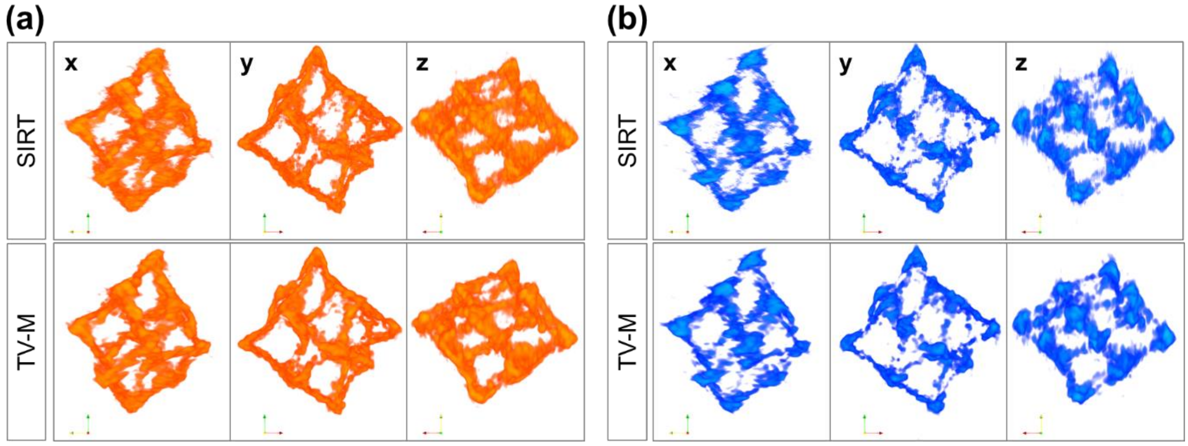

Fig. S8 Comparison of EDS tomography result between SIRT and TV-M algorithm. (a) Ir and (b) Ni atoms.
